# Supplementary material for: Genetic Effects Analysis of QTLs for Rice Grain Size Based on CSSL-Z403 and Its Dissected Single and Dual-Segment Substitution Lines
Source: Int J Mol Sci. 2023 Jul 27;24(15):12013. doi: 10.3390/ijms241512013 (PMC10418668; doi:10.3390/ijms241512013)
Supplement: Supplementary file 1 [file ijms-24-12013-s001.zip › ijms-2481532-supplementary.pdf]

Supplementary Data S1. The main results of QTL identification based on substitution lines (S1–S4; D1–D2) and recipient Xihui18 by one-way ANOVA and LSD multiple comparisons. Between groups represented the variations among all SSSLs (S1–S4), DSSLs (D1 and D2) and recipient Xihui18 comparison for each trait as grain length, grain width, 1000-grain weight and ratio of length to width. Within groups represented variation for errors. LSD represented least significant difference multiple comparison, in which I VAR0001 was Xihui18, J VAR0001 was substitution lines (S1–S4 and D1–D2), respectively, mean difference (I–J) was the difference value between Xihui18 and each substitution line. Sig. represented probability value (p) for each trait difference between each substitution line and Xihui18. When Sig. < 0.05 represented a QTL for a certain trait existed in a SSSL (S1–S4) or DSSL.

Supplementary Data S2. The main results of epistatic interaction between Q_1 (located in "*i*" substitution segment) and Q_2 (located in "*j*" substitution segment) in DSSLs (D1–D2) by two-way ANOVA. Tests of between subjects effects represented Q_1, Q_2 and $Q_1 \times Q_2$ test using DSSL containing both the "*i*" and "*j*" substitution segments and the responding $SSSL_i$ and $SSSL_j$ by two-way ANOVA, in which sig. < 0.05 for Q_1 or Q_2 indicate additive effects of Q_1 or Q_2 existed; sig. > 0.05 for Q_1 or Q_2 indicate no significant additive effects of Q_1 or Q_2 existed and "-" was shown; sig. < 0.05 for $Q_1 \times Q_2$ indicated epistatic effect of Q_1 and Q_2 interaction existed in the DSSL; sig. > 0.05 for $Q_1 \times Q_2$ indicated independent inheritance of Q_1 and Q_2 in the DSSL.
