# Supplementary material for: Genetic Effects Analysis of QTLs for Rice Grain Size Based on CSSL-Z403 and Its Dissected Single and Dual-Segment Substitution Lines
Source: Int J Mol Sci. 2023 Jul 27;24(15):12013. doi: 10.3390/ijms241512013 (PMC10418668; doi:10.3390/ijms241512013)
Supplement: Supplementary file 1 [file ijms-24-12013-s001.zip › Supplement data 1.pdf]

Supplement data 1: One-way ANOVA and LSD multiple comparison for 4 traits between Xihui18 and S1-S4

Grain length(mm)：

ANOVA

	Sum of Squares	df	Mean Square	F	Sig.
Between Groups	5.767	6	.961	54.192	.000
Within Groups	1.117	63	.018		
Total	6.885	69			

Multiple Comparisons

	(I) VAR00001	(J) VAR00001	Mean Difference (I-J)	Std. Error	Sig.	95% Confidence Interval	
						Lower Bound	Upper Bound
LSD	Xihui18	S1	.15400*	.05956	.012	.0350	.2730
		S2	.00711	.05956	.905	-.1119	.1261
		S3	-.46300*	.05956	.000	-.5820	-.3440
		S4	.38000*	.05956	.000	.2610	.4990
		D2	-.24200*	.05956	.000	-.3610	-.1230
		D1	.37700*	.05956	.000	.2580	.4960

Grain width(mm)：

ANOVA

	Sum of Squares	df	Mean Square	F	Sig.
Between Groups	9.860	6	1.643	353.580	.000
Within Groups	.293	63	.005		
Total	10.153	69			

Multiple Comparisons

	(I) VAR00001	(J) VAR00001	Mean Difference (I-J)	Std. Error	Sig.	95% Confidence Interval	
						Lower Bound	Upper Bound
LSD	Xihui18	S1	-1.28000*	.03049	.000	-1.3409	-1.2191
		S2	-.41711*	.03049	.000	-.4780	-.3562
		S3	-.68400*	.03049	.000	-.7449	-.6231
		S4	-.29400*	.03049	.000	-.3549	-.2331
		D1	-.61200*	.03049	.000	-.6729	-.5511
		D2	-.31700*	.03049	.000	-.3779	-.2561

1000-grain weight (g)：

ANOVA

	Sum of Squares	df	Mean Square	F	Sig.
Between Groups	820.332	6	136.722	204.812	.000
Within Groups	37.383	56	.668		
Total	857.715	62			

Multiple Comparisons

	(I) VAR00001	(J) VAR00001	Mean Difference (I-J)	Std. Error	Sig.	95% Confidence Interval	
						Lower Bound	Upper Bound
LSD	Xihui18	S1	-9.296*	.403	.000	-10.10	-8.49
		S2	-.686	.423	.111	-1.53	.16
		S3	-6.526*	.403	.000	-7.33	-5.72
		S4	-.173	.423	.684	-1.02	.67
		D1	-5.908*	.403	.000	-6.71	-5.10
		D2	-.368	.403	.365	-1.17	.44

Ratio of length to width:

ANOVA

	Sum of Squares	df	Mean Square	F	Sig.
Between Groups	6.107	6	1.018	245.058	.000
Within Groups	.262	63	.004		
Total	6.369	69			

Multiple Comparisons

	(I) VAR00003	(J) VAR00003	Mean Difference (I-J)	Std. Error	Sig.	95% Confidence Interval	
						Lower Bound	Upper Bound
LSD	Xihui18	S1	1.08883*	.02882	.000	1.0312	1.1464
		S2	.43191*	.02882	.000	.3743	.4895
		S3	.52890*	.02882	.000	.4713	.5865
		S4	.42783*	.02882	.000	.3702	.4854
		D1	.53041*	.02882	.000	.4728	.5880
		D2	.44843*	.02882	.000	.3908	.5060
