# Supplementary material for: Genetic Effects Analysis of QTLs for Rice Grain Size Based on CSSL-Z403 and Its Dissected Single and Dual-Segment Substitution Lines
Source: Int J Mol Sci. 2023 Jul 27;24(15):12013. doi: 10.3390/ijms241512013 (PMC10418668; doi:10.3390/ijms241512013)
Supplement: Supplementary file 1 [file ijms-24-12013-s001.zip › Supplement data 2.pdf]

**Supplement data2:** Two-way ANOVA of two QTL epistattic interaction in substitution segments of each DSSL

Grain length(mm)：

D1:

**Tests of Between-Subjects Effects**

| Source | Type III Sum of Squares | df | Mean Square | F | Sig. |
|---|---|---|---|---|---|
| Corrected Model | 4.793[a] | 3 | 1.598 | 100.259 | .000 |
| Intercept | 4479.572 | 1 | 4479.572 | 281115.297 | .000 |
| *qGL5* (S1) | .543 | 1 | .543 | 34.069 | .000 |
| Substitution Seg. (S2) | 3.721 | 1 | 3.721 | 233.511 | .000 |
| *qGL5*(S1)×substitution seg.(S2) | .529 | 1 | .529 | 33.197 | .000 |
| Error | .574 | 36 | .016 | | |
| Total | 4484.939 | 40 | | | |
| Corrected Total | 5.367 | 39 | | | |

a. R Squared = .893 (Adjusted R Squared = .884)

D2:

**Tests of Between-Subjects Effects**

| Source | Type III Sum of Squares | df | Mean Square | F | Sig. |
|---|---|---|---|---|---|
| Corrected Model | .807[a] | 3 | .269 | 14.932 | .000 |
| Intercept | 4559.293 | 1 | 4559.293 | 253118.255 | .000 |
| *qGL9* (S3) | .023 | 1 | .023 | 1.253 | .270 |
| *qGL12* (S4) | .378 | 1 | .378 | 21.002 | .000 |
| *qGL9*(S3)×*qGL12*(S4) | .406 | 1 | .406 | 22.541 | .000 |
| Error | .648 | 36 | .018 | | |
| Total | 4560.748 | 40 | | | |
| Corrected Total | 1.455 | 39 | | | |

a. R Squared = .554 (Adjusted R Squared = .517)

Grain width(mm)：

D1:

**Tests of Between-Subjects Effects**

| Source | Type III Sum of Squares | df | Mean Square | F | Sig. |
|---|---|---|---|---|---|
| Corrected Model | 2.355[a] | 3 | .785 | 189.367 | .000 |
| Intercept | 445.623 | 1 | 445.623 | 107486.955 | .000 |
| *qGW5* (S1) | 1.250 | 1 | 1.250 | 301.416 | .000 |
| *qGW7* (S2) | .013 | 1 | .013 | 3.213 | .081 |
| *qGW5*(S1)×*qGW7*(S2) | 1.092 | 1 | 1.092 | 263.470 | .000 |
| Error | .149 | 36 | .004 | | |
| Total | 448.127 | 40 | | | |
| Corrected Total | 2.504 | 39 | | | |

a. R Squared = .940 (Adjusted R Squared = .935)

D2:

**Tests of Between-Subjects Effects**

| Source | Type III Sum of Squares | df | Mean Square | F | Sig. |
|---|---|---|---|---|---|
| Corrected Model | 8.540[a] | 3 | 2.847 | 669.731 | .000 |
| Intercept | 515.883 | 1 | 515.883 | 121376.317 | .000 |
| *qGW9* (S3) | 5.439 | 1 | 5.439 | 1279.696 | .000 |
| *qGW12* (S4) | .158 | 1 | .158 | 37.057 | .000 |
| *qGW9*(S3)×*qGW12*(S4) | 2.943 | 1 | 2.943 | 692.440 | .000 |
| Error | .153 | 36 | .004 | | |
| Total | 524.576 | 40 | | | |
| Corrected Total | 8.693 | 39 | | | |

a. R Squared = .982 (Adjusted R Squared = .981)

1000-grain weight (g)：

D1:

**Tests of Between-Subjects Effects**

| Source | Type III Sum of Squares | df | Mean Square | F | Sig. |
|---|---|---|---|---|---|
| Corrected Model | 507.330[a] | 3 | 169.110 | 604.133 | .000 |
| Intercept | 36302.576 | 1 | 36302.576 | 129688.342 | .000 |
| *qGWT5* (S1) | 449.124 | 1 | 449.124 | 1604.462 | .000 |
| *qGWT7* (S2) | 15.863 | 1 | 15.863 | 56.670 | .000 |
| *qGWT5*(S1)×*qGWT7*(S2) | 35.855 | 1 | 35.855 | 128.088 | .000 |
| Error | 8.678 | 31 | .280 | | |
| Total | 38882.220 | 35 | | | |
| Corrected Total | 516.007 | 34 | | | |

a. R Squared = .983 (Adjusted R Squared = .982)

D2:

**Tests of Between-Subjects Effects**

| Source | Type III Sum of Squares | df | Mean Square | F | Sig. |
|---|---|---|---|---|---|
| Corrected Model | 286.159[a] | 3 | 95.386 | 102.155 | .000 |
| Intercept | 31565.256 | 1 | 31565.256 | 33805.256 | .000 |
| *qGWT9* (S3) | 96.528 | 1 | 96.528 | 103.378 | .000 |
| Substitution Seg. (S4) | 76.557 | 1 | 76.557 | 81.990 | .000 |
| *qGWT9*(S3)× substitution seg.(S4) | 85.676 | 1 | 85.676 | 91.756 | .000 |
| Error | 28.946 | 31 | .934 | | |
| Total | 33137.908 | 35 | | | |
| Corrected Total | 315.105 | 34 | | | |

a. R Squared = .908 (Adjusted R Squared = .899)

Ratio of length to width:

D1:

**Tests of Between-Subjects Effects**

| Source | Type III Sum of Squares | df | Mean Square | F | Sig. |
|---|---|---|---|---|---|
| Corrected Model | 1.707[a] | 3 | .569 | 113.274 | .000 |
| Intercept | 405.833 | 1 | 405.833 | 80776.123 | .000 |
| *qRLW5* (S1) | .754 | 1 | .754 | 149.976 | .000 |
| *qRLW7* (S2) | .301 | 1 | .301 | 59.915 | .000 |
| *qRLW5*(S1)×*qRLW7*(S2) | .653 | 1 | .653 | 129.932 | .000 |
| Error | .181 | 36 | .005 | | |
| Total | 407.721 | 40 | | | |
| Corrected Total | 1.888 | 39 | | | |

a. R Squared = .904 (Adjusted R Squared = .896)

D2：

**Tests of Between-Subjects Effects**

| Source | Type III Sum of Squares | df | Mean Square | F | Sig. |
|---|---|---|---|---|---|
| Corrected Model | 6.030[a] | 3 | 2.010 | 647.730 | .000 |
| Intercept | 365.723 | 1 | 365.723 | 117858.851 | .000 |
| *qRLW9* (S3) | 3.534 | 1 | 3.534 | 1138.975 | .000 |
| *qRLW12* (S4) | .040 | 1 | .040 | 12.994 | .001 |
| *qRLW9*(S3)×*qRLW12*(S4) | 2.455 | 1 | 2.455 | 791.221 | .000 |
| Error | .112 | 36 | .003 | | |
| Total | 371.864 | 40 | | | |
| Corrected Total | 6.142 | 39 | | | |

a. R Squared = .982 (Adjusted R Squared = .980)
